# Supplementary material for: Genetic and Epigenetic Regulation in Lingo-1: Effects on Cognitive Function and White Matter Microstructure in a Case-Control Study for Schizophrenia
Source: Int J Mol Sci. 2023 Oct 26;24(21):15624. doi: 10.3390/ijms242115624 (PMC10648795; doi:10.3390/ijms242115624)
Supplement: Supplementary file 1 [file ijms-24-15624-s001.zip › ijms-2657001-supplementary.pdf]

Supplementary Methods

Psychometric Testing

All recruited subjects were subjected to thorough psychometric testing to assess the extent of psychiatric illness in the schizophrenia population, in addition to excluding the presence of mental disorders in the control group. The battery of psychometric testing undertaken by all subjects included: the Psychosis Screener (from the Western Australian Family Study of Schizophrenia), the Diagnostic Interview for Psychosis (ASRB modification[1]), the Scale for the Assessment of Negative Symptoms (SANS) for symptom severity, the Global Assessment of Functioning (GAF) for general functioning, the International Personality Disorder Examination Screening Questionnaire (ICD-10 Module), the Childhood Adversity Questionnaire, and the Schizotypal Personality Questionnaire (SPQ).

Genotyping Assay Design and Quality Control

PCR and extension primer design, selection and multiplexing were performed using MassARRAY® Designer Software (Sequenom, Inc., San Diego, CA). Control samples were run in parallel with the case-control population, allowing for accurate evaluation and quality control checks of each of the steps involved (PCR amplification, Shrimp Alkaline Phosphatase (SAP) treatment, extension PCR, nano-dropping to check product concentrations, nano-dispensing of samples onto the chip, and instrument performance). Additionally, only samples which had a call rate of ≥80% were included in the analysis for this study. Any samples yielding less than 80% were omitted from analysis.

Sequenom Methylation Assay

High-throughput bisulfite conversion of denatured DNA samples was performed using an EZ-DNA Methylation-Gold™ Kit (Zymo Research, Irvine, CA, USA). Bisulfite converted DNA was subjected to quality-control quantitative PCR (qPCR) assays to assess the completeness of the bisulfite conversion and the amount of converted DNA in each sample. Bisulfite converted DNA within the *Lingo-1* loci of interest was then amplified with bisulfite specific PCR primers, followed by removal of unused dNTPs using a SAP treatment, before a single-stranded nucleic acid cleavage step. *Lingo-1* specific sequencing primers to amplify the regions of interest were added to each treated sample to allow fragments to be sequenced using MALDI-TOF MS to detect the presence of methylated cytosines.

MRI Acquisition and Image Processing

A large subset of both control and schizophrenia subjects also underwent structural magnetic resonance imaging brain scans to obtain diffusion weighted images at 5 different sites across Australia, with all sites utilizing the same MRI system and acquisition sequence. Scanners used were Siemens Avanto 1.5 Tesla scanners (Siemens, Erlangen, Germany). Scans were acquired using a spin-echo EPI sequence with the following parameters being applied: b-value, 1000 s/mm^2; 65 consecutive axial slices of thickness 2.4 mm; 104 × 104 image matrix with an in-plane voxel resolution of 2.4 × 2.4 mm; field of view, 25 × 25 cm; repetition time, 8.4 s; echo time, 88 ms; flip angle, 90 degrees. T1-weighted brain anatomy images were also acquired using an optimized magnetization-prepared rapid acquisition gradient echo, and T2-weighted (i.e., b=0) brain volumes were acquired prior to the acquisition of the diffusion-weighted volumes. The diffusion images were corrected for head movements and EPI distortions using affine transformations to each individuals T2-weighted (b=0) volume. Whole-brain fractional anisotropy images were generated for each participant by fitting the diffusion tensor model to each voxel using least squares estimation. Fractional anisotropy is an index of white matter structure that reflects axonal density, diameter and myelination. In this study, an average fractional anisotropy value was determined for each subject by averaging fractional anisotropy across all voxels comprising a liberal white matter mask. The white matter mask was

warped to each individual based on the inverse of the warp from the individual's fractional anisotropy image to MNI standard space. These steps were performed with the FMRIB Software Library (FSL). Each fractional anisotropy map was inspected for gross abnormalities as a quality control measure.

Supplementary Results

Supplementary Table S1. Complete results of MANOVAs performed to determine Diagnosis, Genotype, and Diagnosis x Genotype effects on methylation status, in all subjects (both sexes) and male and female subjects individually. Note that Least Significant Difference (LSD) post-hoc tests are shown only for the significant results highlighted in bold.

		**(a) Lingo-1 rs3144 (TT:TC:CC)**			**(b) Lingo-1 rs3144 (TT:TC/CC)**		
		all	male	female	all	male	female
**CpG1**	Diagnosis	F=0.198, p=0.657	F=0.256, p=0.614	F=0.069, p=0.794	F=0.046, p=0.831	F=0.014, p=0.905	F=0.000013, p=0.997
	Genotype	F=1.767, p=0.173	F=1.066, p=0.347	F=0.669, p=0.515	F=1.852, p=0.175	F=1.616, p=0.205	F=0.057, p=0.812
	Diagnosis x Genotype	F=1.098, p=0.335	F=1.061, p=0.348	F=0.257, p=0.774	F=1.795, p=0.181	F=1.966, p=0.163	F=0.100, p=0.753
	LSD post-hoc	-	-	-	-	-	-
		-	-	-	-	-	-
**CpG2**	Diagnosis	F=0.744, p=0.389	F=3.077, p=0.081	F=0.894, p=0.347	F=0.405, p=0.525	F=1.602, p=0.207	F=0.701, p=0.405
	Genotype	F=0.597, p=0.551	F=0.533, p=0.588	F=0.250, p=0.779	F=1.242, p=0.266	F=1.200, p=0.275	F=0.465, p=0.497
	Diagnosis x Genotype	F=0.174, p=840	F=1.447, p=0.238	F=0.666, p=0.517	F=0.001, p=0.977	F=0.210, p=0.647	F=0.438, p=0.510
	LSD post-hoc	-	-	-	-	-	-
		-	-	-	-	-	-
**CpG3**	Diagnosis	F=1.013, p=0.315	F=1.083, p=0.299	F=0.119, p=731	F=0.077, p=0.782	F=0.113, p=0.737	F=0.000195, p=989
	Genotype	F=1.088, 0.338	F=0.721, p=0.487	F=1.039, p=0.359	F=0.263, p=0.609	F=0.724, p=0.396	F=0.122, p=0.728
	Diagnosis x Genotype	F=0.975, p=0.379	F=0.850, p=0.429	F=0.466, p=0.629	F=1.216, p=0.271	F=0.600, p=0.440	F=0.825, p=0.366
	LSD post-hoc	-	-	-	-	-	-
		-	-	-	-	-	-
**CpG4**	Diagnosis	F=0.930, p=0.336	F=0.033, p=0.857	F=2.624, p=0.109	F=0.152, p=0.697	F=0.044, p=0.833	F=0.799, p=0.374
	Genotype	F=0.793, p=0.454	F=1.207, p=0.302	F=0.047, p=0.954	F=0.047, p=0.828	F=0.129, 0.720	F=0.001, p=0.977
	Diagnosis x Genotype	F=0.877, p=0.417	F=0.324, p=0.723	F=1.136, p=0.326	F=1.506, p=0.221	F=0.605, p=0.720	F=0.771, p=0.382
		-	-	-	-	-	-

	LSD post-hoc	-	-	-	-	-	-
**CpG5**	Diagnosis	F=2.683, p=0.103	F=2.478, p=0.117	F=0.483, p=0.489	**F=4.622, p=0.032**	F=2.966, p=0.087	F=1.626, p=0.206
	Genotype	F=0.026, p=0.957	F=0.003, p=0.997	F=0.131, p=0.877	F=0.026, p=0.871	F=0.006, p=0.937	F=0.035, p=0.852
	Diagnosis x Genotype	F=1.618, p=0.200	F=0.224, p=0.800	F=2.926, p=0.059	F=0.188, p=0.665	F=0.177, p=0.675	F=0.015, p=0.903
	LSD post-hoc	-	-	-	**HC>SZ, p=0.032**	-	-
		-	-	-	-	-	-
**CpG6**	Diagnosis	F=2.574, p=0.110	F=1.079, p=0.300	F=1.500, p=0.224	F=0.940, p=0.333	F=0.359, p=0.550	F=0.671, p=0.415
	Genotype	F=1.116, 0.329	F=1.623, p=0.200	F=1.502, p=0.229	F=1.098, p=0.296	F=0.031, p=0.861	F=2.325, p=0.131
	Diagnosis x Genotype	F=0.803, p=0.449	F=0.860, p=0.425	F=0.808, p=0.449	F=1.140, p=0.287	F=1.526, p=0.218	F=0.000067, p=0.993
	LSD post-hoc	-	-	-	-	-	-
		-	-	-	-	-	-
**CpG7**	Diagnosis	F=1.713, p=0.192	F=2.246, p=0.136	F=0.45, p=0.832	F=2.947, p=0.087	F=3.573, p=0.060	F=0.014, p=0.907
	Genotype	F=0.044, p=0.957	F=0.817, p=0.443	F=1.871, p=0.161	F=0.068, p=0.794	F=0.572, p=0.451	F=3.010, p=0.087
	Diagnosis x Genotype	F=1.178, p=0.310	F=2.783, p=0.065	F=0.498, p=0.610	F=2.322, p=0.129	**F=5.054, p=0.026**	F=0.736, p=0.393
	LSD post-hoc	-	-	-	-	HC:TC/CC>TT, p=0.427	-
		-	-	-	-	SZ:TT>TC/CC, p=0.483	-
**CpG8**	Diagnosis	F=0.579, p=0.447	F=0.816, p=0.368	F=0.079, p=0.779	F=0.289, p=0.591	F=0.176, p=0.676	F=0.332, p=0.566
	Genotype	**F=3.222, p=0.041**	F=2.193, p=0.115	F=2.496, p=0.089	F=0.539, p=0.464	F=2.001, p=0.159	F=1.159, p=0.285
	Diagnosis x Genotype	F=0.766, p=0.466	F=1.736, p=0.179	F=0.235, p=0.791	F=1.653, p=0.200	F=3.692, p=0.056	F=0.370, p=0.545
	LSD post-hoc	**TT>CC, p=0.033**	-	-	-	-	-
		**TC>CC, p=0.012**	-	-	-	-	-
**CpG9**	Diagnosis	F=0.603, p=0.438	F=0.162, p=0.688	F=2.194, p=0.143	F=1.929, p=0.166	F=0.575, p=0.449	F=1.352, p=0.248
	Genotype	F=1.338, p=0.264	F=0.621, p=0.539	F=0.476, p=0.623	F=2.342, p=0.127	F=1.326, p=0.251	F=0.733, p=0.395
	Diagnosis x Genotype	F=0.840, p=0.433	F=2.455, p=0.089	F=0.144, p=0.866	F=0.345, p=0.558	F=1.095, p=0.297	F=0.112, p=0.738
	LSD post-hoc	-	-	-	-	-	-
		-	-	-	-	-	-
**CpG10**	Diagnosis	F=2.043, p=0.154	F=0.394, p=0.531	F=3.660, p=0.059	F=2.287, p=0.132	F=0.029, p=0.866	**F=6.513, p=0.013**

	Genotype	F=1.430, p=0.241	F=1.453, p=0.237	F=1.359, p=0.263	F=2.923, p=0.088	F=2.596, p=0.109	F=0.630, p=0.430
	Diagnosis x Genotype	F=0.258, p=0.773	F=1.322, p=0.269	F=2.332, p=0.104	F=0.397, p=0.529	F=0.204, p=0.652	F=0.569, p=0.453
	LSD post-hoc	-	-	-	-	-	**HC>SZ, p=0.013**
		-	-	-	-	-	-

Supplementary Table S2. Summary of results from Pearson's Correlations performed to determine interactions between cognitive performance measures and methylation status, in all subjects, healthy controls, schizophrenia subjects, and male and female healthy control and schizophrenia subjects individually. Significant correlations shown in bold.

		**Cpg1**	**CpG2**	**CpG3**	**CpG4**	**CpG5**	**CpG6**	**CpG7**	**CpG8**	**CpG9**	**CpG10**
**WTAR standard score (US standardization)**	***all subjects***	r=0.007, p=0.879	r=0.038, p=0.411	r=0.027, p=0.559	r=0.025, p=0.590	r=0.062, p=0.165	r<0.001, p=1.000	r=-0.023, p=0.604	r=-0.068, p=0.132	r=0.006, p=0.887	r=0.066, p=0.145
	healthy control	r=0.017, p=0.817	r=0.036, p=0.570	r=0.030, p=0.632	r=-0.023, p=0.721	r=-0.004, p=0.951	r=0.042, p=0.502	r=0.069, p=0.274	r=-0.066, p=0.295	r=-0.107, p=0.087	r=0.023, p=0.714
	schizophrenia	r=-0.005, p=0.941	r=0.076, p=0.252	r=-0.003, p=0.969	r=0.047, p=0.478	r=0.060, p=0.358	r=-0.028, p=0.668	r=-0.089, p=0.169	r=-0.066, p=0.309	r=0.110, p=0.090	r=0.068, p=0.295
	male healthy control	r=-0.010, p=0.903	r=0.015, p=0.858	r=0.024, p=0.768	r=-0.003, p=0.973	r=-0.003, p=0.968	r=0.029, p=0.711	r=0.044, p=0.575	r=-0.067, p=0.394	r=-0.093, p=0.240	r=-0.011, p=0.888
	female healthy control	r=0.062, p=0.594	r=0.072, p=0.498	r=0.045, p=0.675	r=-0.069, p=0.521	r=-0.004, p=0.969	r=0.076, p=0.474	r=0.108, p=0.304	r=-0.055, p=0.602	r=-0.130, p=0.216	r=0.092, p=0.381
	male schizophrenia	r=0.005, p=0.955	r=0.089, p=0.265	r=0.039, p=0.621	r=0.027, p=0.732	r=0.083, p=0.288	r=-0.003, p=0.971	r=-0.0132, p=0.091	r=-0.108, p=0.168	r=0.127, p=0.104	r=0.040, p=0.608
	female schizophrenia	r=-0.036, p=0.792	r=0.104, p=0.390	r=-0.116, p=0.332	r=0.115, p=0.347	r=-0.021, p=0.857	r=-0.069, p=0.561	r=0.014, p=0.903	r=0.045, p=0.706	r=0.056, p=0.637	r=0.159, p=0.176
**WASI (Intelligence scale)**	***all subjects***	r=-0.020, p=0.690	r=0.005, p=0.915	r=0-0.001, p=0.982	r=-0.034, p=0.466	r=0.075, p=0.094	r=-0.065, p=0.149	r=-0.016, p=0.726	r=-0.030, p=0.509	r=0.021, p=0.639	r=0.011, p=0.803
	healthy control	r=-0.017, p=0.303	r=0.017, p=0.795	r<0.001, p=0.996	r=-0.084, p=0.190	r=-0.077, p=0.219	r=-0.013, p=0.836	r=-0.006, p=0.930	r=-0.008, p=0.896	r=-0.018, p=0.777	r=-0.072, p=0.249
	schizophrenia	r=0.003, p=0.964	r=0.060, p=0.367	r=-0.057, p=0.389	r=-0.045, p=0.495	r=0.097, p=0.133	r=-0.155, p=0.077	r=-0.016, p=0.805	r=-0.037, p=0.570	r=0.073, p=0.262	r=0.004, p=0.945
	male healthy control	r=-0.125, p=0.141	r=0.007, p=0.933	r=0.009, p=0.907	r=-0.094, p=0.243	r=-0.082, p=0.297	r=-0.001, p=0.992	r=-0.039, p=0.625	r=-0.067, p=0.393	r=-0.022, p=0.782	r=-0.077, p=0.331
	female healthy control	r=0.059, p=0.615	r=0.044, p=0.682	r=-0.020, p=0.851	r=-0.047, p=0.660	r=-0.073, p=0.492	r=-0.048, p=0.649	r=0.079, p=0.456	r=0.087, p=0.410	r=-0.030, p=0.779	r=-0.033, p=0.755
	male schizophrenia	r<0.000, p=0.998	r=-0.012, p=0.881	r=-0.083, p=0.291	r=-0.117, p=0.141	r=0.071, p=0.364	r=-0.080, p=0.305	r=-0.014, p=0.862	r=-0.034, p=0.666	r=0.060, p=0.444	r=-0.033, p=0.677
	female schizophrenia	r=0.014,	**r=0.260,**	r=0.012,	r=0.157,	r=0.152,	r=-0.172,	r=-0.013,	r=-0.040,	r=0.079,	r=0.109,

		Cpg1	CpG2	CpG3	CpG4	CpG5	CpG6	CpG7	CpG8	CpG9	CpG10
		p=0.918	**p=0.028**	p=0.920	p=0.197	p=0.195	p=0.143	p=0.910	p=0.706	p=0.505	p=0.354
**LNS (working memory)**	*all subjects*	r=0.035, p=0.477	r=0.015, p=0.743	r=-0.036, p=0.434	r=-0.002, p=0.969	r=0.003, p=0.949	r=0.010, p=0.817	r=0.035, p=0.436	r=-0.026, p=0.567	r=-0.043, p=0.345	r=-0.014, p=0.754
	healthy control	r=-0.014, p=0.840	r=0.065, p=0.312	r=-0.040, p=0.529	r=0.084, p=0.192	r=-0.075, p=0.234	r=0.058, p=0.358	**r=0.148, p=0.018**	r=-0.061, p=0.334	r=-0.042, p=0.507	r=-0.082, p=0.192
	schizophrenia	r=0.089, p=0.207	r=0.028, p=0.673	r=-0.097, p=0.138	**r=-0.141, p=0.033**	r=-0.037, p=0.570	r=-0.025, p=0.696	r=-0.057, p=0.380	r=0.037, p=0.567	r=-0.043, p=0.510	r=-0.022, p=0.734
	male healthy control	r=0.032, p=0.709	r=0.011, p=0.890	r=0.018, p=0.819	r=0.085, p=0.289	r=-0.071, p=0.366	r=-0.009, p=0.907	r=0.153, p=0.052	r=-0.060, p=0.448	r=-0.073, p=0.353	r=-0.085, p=0.279
	female healthy control	r=-0.095, p=414	r=0.171, p=0.108	r=-0.161, p=0.128	r=0.103, 0.336	r=-0.087, p=0.410	r=0.179, p=0.090	r=0.173, p=0.100	r=-0.089, p=0.410	r=-0.001, p=0.989	r=-0.041, p=0.698
	male schizophrenia	r=0.102, p=0.220	r=-0.025, p=0.754	r=-0.134, p=0.090	**r=-0.224, p=0.004**	r=-0.032, p=0.686	r=0.059, p=0.449	r=-0.139, p=0.074	r=-0.013, p=0.864	r=-0.112, p=0.151	r=-0.038, p=0.631
	female schizophrenia	r=0.053, p=0.697	r=0.159, p=0.184	r=-0.005, p=0.968	r=0.097, p=0.426	r=-0.055, p=0.640	r=-0.193, p=0.099	r=0.126, p=0.285	r=0.165, p=0.167	r=0.097, p=0.410	r=0.018, p=0.882
**COWAT (executive functioning)**	*all subjects*	r=-0.030, p=0.535	r=-0.009, p=0.847	r=0.019, p=0.677	r=0.020, p=0.662	**r=0.104, p=0.021**	r=-0.052, p=0.250	r=0.007, p=0.883	r=-0.056, p=0.212	r=0.019, p=0.671	r=0.034, p=0.457
	healthy control	r=-0.070, p=0.309	r=0.003, p=0.957	r=0.019, p=0.762	r=0.045, p=0.485	r=0.073, p=0.249	r=-0.010, p=0.875	r=0.031, p=0.624	r=-0.110, p=0.080	r=-0.036, p=0.571	r=-0.015, p=0.814
	schizophrenia	r=0.001, p=0.993	r=0.017, p=0.802	r=-0.015, p=0.825	r=-0.031, p=0.644	r=0.070, p=0.283	r=-0.093, p=0.150	r=-0.007, p=0.917	r=0.016, p=0.804	r=0.088, p=0.176	r=0.036, p=0.577
	male healthy control	r=-0.072, p=0.397	r=0.040, p=0.624	r=0.038, p=0.640	r=0.036, p=0.652	r=0.089, p=0.259	r=-0.026, p=0.742	r=0.042, p=0.593	r=-0.125, p=0.112	r=-0.068, p=0.389	r=-0.028, p=0.724
	female healthy control	r=-0.066, p=0.569	r=-0.060, p=0.571	r=-0.021, p=0.841	r=0.077, p=0.474	r=0.035, p=0.742	r=0.015, p=0.884	r=0.023, p=0.825	r=-0.089, p=0.397	r=0.019, p=0.855	r=0.039, p=0.715
	male schizophrenia	r=0.021, p=0.806	r=0.102, p=0.202	r=-0.044, p=0.574	r=-0.063, p=0.429	r=0.120, p=0.122	r=-0.052, p=0.510	r=-0.019, p=0.809	r=0.063, p=0.418	r=0.073, p=0.348	r=0.030, p=0.701
	female schizophrenia	r=-0.051, p=0.707	r=-0.090, p=0.454	r=0.055, p=0.646	r=0.055, p=0.655	r=-0.073, p=0.538	r=-0.155, p=0.187	r=0.027, p=0.818	r=-0.084, p=0.485	r=0.091, p=0.439	r=0.082, p=0.486
**RBANS TOTAL (Sum of all**	*all subjects*	r=0.017, p=0.723	r=-0.030, p=0.520	r=0.18, p=0.686	r=0.006, p=0.898	r=0.066, p=0.140	r=-0.030, p=0.513	r=0.019, p=0.668	r=-0.017, p=0.704	r=-0.009, p=0.845	r=0.049, p=0.273

		Cpg1	CpG2	CpG3	CpG4	CpG5	CpG6	CpG7	CpG8	CpG9	CpG10
**RBANs tests scores)**	*healthy control*	r=0.008, p=0.902	r=-0.028, p=0.666	r=0.020, p=0.757	r=0.012, p=0.851	r=0.008, p=0.897	r=-0.001, p=0.983	r=0.097, p=0.124	r=-0.010, p=0.875	r=-0.016, p=0.796	r=-0.009, p=0.881
	schizophrenia	r=0.021, p=0.766	r=0.040, p=0.542	r=-0.052, p=0.432	r=-0.055, p=0.408	r=-0.014, p=0.830	r=-0.056, p=0.388	r=-0.020, p=0.762	r=-0.001, p=0.991	r=0.013, p=0.841	r=0.026, p=0.692
	male healthy control	r=-0.031, p=0.721	r=-0.021, p=0.794	r=0.090, p=0.261	r=0.017, p=0.836	r=-0.020, p=0.801	r=0.009, p=0.908	r=0.103, p=0.192	r=-0.052, p=0.508	r=-0.015, p=0.847	r=0.048, p=0.546
	female healthy control	r=0.088, p=0.451	r=-0.044, p=0.681	r=-0.123, p=0.246	r=-0.003, p=0.977	r=0.068, p=0.520	r=-0.019, p=0.857	r=0.081, p=0.444	r=0.090, p=0.395	r=-0.014, p=0.893	r=-0.157, p=0.136
	male schizophrenia	r=0.026, p=0.753	r=-0.017, p=0.832	r=-0.086, p=0.277	**r=-0.156, p=0.049**	r=-0.039, p=0.621	r=0.028, p=0.717	r=-0.079, p=0.311	r=-0.019, p=0.810	r=-0.006, p=0.937	r=0.034, p=0.666
	female schizophrenia	r=0.012, p=0.928	**r=0.236, p=0.047**	r=0.021, p=0.860	r=0.181, p=0.137	r=0.005, p=0.968	r=-0.172, p=0.142	r=0.104, p=0.377	r=0.048, p=0.687	r=0.001, p=0.994	r=0.070, p=0.556
**RBANS immediate memory**	*all subjects*	r=0.048, p=0.324	r=-0.059, p=0.197	r=0.026, p=0.565	r=0.009, p=0.847	r=-0.003, p=0.943	r=-0.065, p=0.153	r=0.043, p=0.336	r=-0.009, p=0.849	r=0.003, p=0.950	r=0.031, p=0.493
	healthy control	r=0.056, p=0.410	r=-0.071, p=0.269	r=0.062, p=0.333	r=-0.021, p=0.851	r=-0.060, p=0.340	r=-0.032, p=0.616	**r=0.187, p=0.003**	r=-0.009, p=0.882	r=0.012, p=0.849	r=-0.029, p=0.640
	schizophrenia	r=0.043, p=0.548	r=-0.009, p=0.888	r=-0.054, p=0.413	r=-0.007, p=0.913	r=-0.088, p=0.174	r=-0.105, p=0.107	r=-0.039, p=0.544	r=0.015, p=0.817	r=0.004, p=0.947	r=0.007, p=0.911
	male healthy control	r=0.058, p=0.497	r=-0.081, p=0.319	r=0.097, p=0.225	r=-0.012, p=0.886	r=-0.063, p=0.426	r=-0.022, p=0.781	r=0.150, p=0.057	r=-0.051, p=0.521	r=-0.056, p=0.481	r=0.047, p=0.556
	female healthy control	r=0.026, p=0.826	r=-0.068, p=0.526	r=-0.003, p=0.980	r=-0.059, p=0.581	r=-0.052, p=0.623	r=-0.038, p=0.720	**r=0.238, p=0.022**	r=0.096, p=0.362	r=0.151, p=0.151	r=-0.233, p=0.026
	male schizophrenia	r=0.074, p=0.376	r=-0.044, p=0.582	r=-0.033, p=0.682	r=-0.075, p=0.345	r=-0.145, p=0.063	r=0.024, p=0.761	r=-0.031, p=0.693	r=-0.034, p=0.663	r=0.009, p=0.910	r=0.009, p=0.908
	female schizophrenia	r=-0.036, p=0.792	r=0.131, p=0.277	r=-0.100, p=0.403	r=0.162, p=0.184	r=0.006, p=0.961	**r=-0.309, p=0.007**	r=-0.041, p=0.727	r=0.134, p=0.262	r=-0.046, p=0.696	r=0.048, p=0.683
**RBANS constructional memory**	*all subjects*	r=-0.002, p=0.966	r=0.002, p=0.972	r=-0.011, p=0.817	r=-0.015, p=0.750	r=0.062, p=0.166	r=0.008, p=0.860	r=-0.067, p=0.138	r<0.001, p=0.997	r=0.009, p=0.842	r=0.018, p=0.693
	healthy control	r=-0.031, p=0.648	r=0.026, p=0.687	r=-0.007, p=0.911	r=-0.042, p=0.514	r=0.059, p=0.349	r=0.016, p=0.797	r=-0.108, p=0.085	r=0.056, p=0.376	r=-0.004, p=944	r=-0.024, p=0.701
	schizophrenia	r=0.018,	r=0.020,	r=-0.059,	r=-0.021,	r=-0.015,	r=0.009,	r=-0.027,	r=-0.050,	r=0.031,	r=0.004,

		Cpg1	CpG2	CpG3	CpG4	CpG5	CpG6	CpG7	CpG8	CpG9	CpG10
		p=0.800	p=0.761	p=0.369	p=0.758	p=0.822	p=0.885	p=0.683	p=0.441	p=0.633	p=0.951
	male healthy control	r=-0.053, p=0.535	r=0.046, p=0.566	r=0.014, p=0.858	r=-0.026, p=0.750	r=0.033, p=0.673	r=0.058, p=0.466	r=-0.102, p=0.194	r=-0.047, p=0.550	r=0.048, p=0.542	r=0.002, p=0.980
	female healthy control	r=0.062, p=0.592	r=0.021, p=0.845	r=-0.050, p=0.637	r=-0.024, p=0.824	r=0.100, p=0.344	r=-0.087, p=0.415	r=-0.064, p=0.542	r=0.204, p=0.051	r=-0.136, p=0.197	r=-0.002, p=0.987
	male schizophrenia	r=-0.010, p=0.906	r=-0.057, p=0.480	r=-0.125, p=0.114	r=-0.063, p=0.423	r=-0.046, p=0.556	r=-0.016, p=0.842	r=-0.083, p=0.290	r=-0.047, p=0.546	r=-0.032, p=0.685	r=-0.017, p=0.833
	female schizophrenia	r=0.098, p=0.470	r=0.141, p=0.241	r=0.103, p=0.389	r=0.096, p=0.431	r=0.084, p=0.477	r=0.047, p=0.691	r=0.084, p=0.476	r=-0.063, p=0.596	r=0.180, p=0.124	r=-0.044, p=0.708
**RBANS language**	*all subjects*	r=0.014, p=0.775	r=0.015, p=0.744	r=0.017, p=0.716	r=0.042, p=0.365	r=0.063, p=0.163	r=-0.029, p=0.527	r=-0.014, p=0.753	**r=-0.096, p=0.034**	r=-0.010, p=0.823	r=0.098, p=0.030
	healthy control	r=-0.017, p=0.800	r=0.051, p=0.429	r=-0.029, p=0.649	r=0.087, p=0.175	r=0.001, p=0.982	r=0.013, p=0.841	r=0.055, p=0.385	**r=-0.129, p=0.040**	r=-0.053, p=0.395	r=0.109, p=0.083
	schizophrenia	r=0.036, p=0.614	r=0.033, p=0.622	r=0.014, p=0.836	r=-0.034, p=0.613	r=0.029, p=0.653	r=-0.067, p=0.305	r=-0.068, p=0.296	r=-0.061, p=0.350	r=0.040, p=0.543	r=0.038, p=0.563
	male healthy control	r=-0.087, p=0.310	r=0.045, p=0.579	r=0.053, p=0.512	r=0.064, p=0.427	r=-0.018, p=0.820	r=-0.091, p=0.249	r=0.086, p=0.276	**r=-0.158, p=0.044**	r=-0.102, p=0.195	r=0.105, p=0.182
	female healthy control	r=0.031, p=0.793	r=0.031, p=0.769	r=-0.171, p=0.105	r=0.069, p=0.523	r=0.049, p=0.641	**r=0.244, p=0.020**	r=-0.079, p=0.455	r=-0.012, p=0.913	r=0.086, p=0.415	r=0.023, p=0.829
	male schizophrenia	r=0.012, p=0.884	r=0.080, p=0.317	r=-0.004, p=0.964	r=-0.085, p=0.286	r=0.068, p=0.384	r=-0.025, p=0.751	**r=-0.164, p=0.036**	r=-0.049, p=0.535	r=0.054, p=0.489	r=0.052, p=0.507
	female schizophrenia	r=0.086, p=0.525	r=0.051, p=0.675	r=0.049, p=0.680	r=0.068, p=0.579	r=-0.078, p=0.511	r=-0.104, p=0.378	r=0.092, p=0.437	r=-0.077, p=0.520	r=-0.029, p=0.806	r=0.072, p=0.542
**RBANS attention**	*all subjects*	r=0.031, p=0.532	r=0.027, p=0.559	r=0.008, p=0.862	r=0.047, p=0.313	r=0.079, p=0.080	r=-0.002, p=0.961	r=0.065, p=0.148	r=-0.012, p=0.788	r=-0.023, p=0.610	r=0.028, p=0.531
	healthy control	r=-0.006, p=0.935	r=0.022, p=0.735	r=-0.017, p=0.794	r=0.099, p=0.121	r=-0.010, p=0.868	r=0.003, p=0.967	r=0.109, p=0.082	r=-0.026, p=0.676	r=-0.054, p=0.391	r=-0.020, p=0.745
	schizophrenia	r=0.062, p=0.379	r=0.112, p=0.092	r=-0.033, p=0.616	r=-0.039, p=0.560	r=0.052, p=0.423	r=0.006, p=0.931	r=0.070, p=0.279	r=0.028, p=0.667	r=0.007, p=0.991	r=-0.009, p=0.893
	male healthy control	r=-0.019, p=0.825	r=0.004, p=0.965	r=0.077, p=0.336	r=0.102, p=0.205	r=-0.022, p=0.782	r=0.037, p=0.640	r=0.077, p=0.328	r=0.035, p=0.655	r=-0.022, p=0.779	r=0.032, p=0.686

		Cpg1	CpG2	CpG3	CpG4	CpG5	CpG6	CpG7	CpG8	CpG9	CpG10
	female healthy control	r=0.023, p=0.844	r=0.051, p=0.632	**r=-0.208, p=0.047**	r=0.095, p=0.375	r=0.015, p=0.890	r=-0.068, p=0.519	r=0.172, p=0.102	r=-0.162, p=0.123	r=-0.114, p=0.279	r=-0.160, p=0.128
	male schizophrenia	r=0.122, p=0.146	r=0.068, p=0.398	r=-0.070, p=0.380	r=-0.130, p=0.102	r=0.078, p=0.319	r=0.072, p=0.357	r=0.028, p=0.718	r=0.026, p=0.744	r=0.008, p=0.919	r=-0.034, p=0.660
	female schizophrenia	r=-0.068, p=0.616	**r=0.275, p=0.020**	r=0.043, p=0.721	r=0.178, p=0.143	r=-0.035, p=0.770	r=-0.0.81, p=0.495	r=0.167, p=0.154	r=-0.045, p=0.704	r=-0.039, p=0.744	r=0.082, p=0.489
**RBANS delayed memory**	*all subjects*	r=-0.018, p=0.709	r=-0.094, p=0.042	r=0.011, p=0.811	r=-0.076, p=0.101	r=0.050, p=0.264	r=-0.015, p=0.741	r=0.008, p=0.856	r=0.030, p=0.503	r=-0.005, p=0.914	r=0.043, p=0.337
	healthy control	r=0.042, p=0.538	r=-0.103, p=0.107	r=0.044, p=0.490	r=-0.103, p=0.106	r=0.031, p=0.622	r<0.001, p=0.998	r=0.061, p=0.335	r=0.037, p=0.558	r=0.024, p=0.701	r=-0.029, p=0.649
	schizophrenia	r=-0.086, p=0.227	r=-0.061, p=0.356	r=-0.061, p=0.353	r=-0.115, p=0.083	r=-0.029, p=0.650	r=-0.020, p=0.756	r=-0.013, p=0.847	r=0.057, p=0.385	r=-0.022, p=0.737	r=0.038, p=0.557
	male healthy control	r=-0.011, p=0.896	r=-0.054, p=0.505	r=0.033, p=0.682	r=-0.118, p=0.143	r=-0.011, p=0.893	r=0.032, p=0.685	r=0.104, p=0.187	r=-0.037, p=0.635	r=0.030, p=0.700	r=-0.003, p=0.965
	female healthy control	r=0.180, p=0.120	r=-0.200, p=0.059	r=0.068, p=0.521	r=-0.080, p=0.455	r=0.122, p=0.248	r=-0.068, p=0.520	r=-0.022, p=0.832	**r=0.207, p=0.048**	r=0.009, p=0.929	r=-0.089, p=0.401
	male schizophrenia	r=-0.099, p=0.237	r=-0.081, p=0.310	r=-0.066, p=0.408	**r=-0.179, p=0.024**	r=-0.060, p=0.443	r=0.048, p=0.542	r=-0.043, p=0.580	r=0.040, p=0.607	r=-0.075, p=0.338	r=0.056, p=0.474
	female schizophrenia	r=-0.046, p=0.733	r=0.084, p=0.489	r=-0.050, p=0.676	r=0.076, p=0.535	r=0.014, p=0.903	r=-0.144, p=0.220	r=0.086, p=0.464	r=0.125, p=0.296	r=0.037, p=0.755	r=0.065, p=0.581

Supplementary Table S3. Summary of results oneway ANOVA performed to determine differences in methylation status between healthy controls, and schizophrenia subjects classified as being cognitively spared (CS) or as having a generalized cognitive deficit (CD) in all subjects, and male and female subjects separately. Least Significant Difference (LSD) post-hoc tests are shown for the significant results highlighted in bold.

	Cpg1	CpG2	CpG3	CpG4	CpG5	CpG6	CpG7	CpG8	CpG9	CpG10
all	F=0.277, p=0.758	F=0.807, p=0.447	F=1.019, p=0.362	F=1.246, p=0.289	**F=3.021, p=0.050**	F=0.043, p=0.958	F=0.010, p=0.990	F=0.255, p=0.775	F=0.883, p=0.414	F=1.345, p=0.261

LSD post-hoc	-	-	-	-	**HC>CD, p=0.023**	-	-	-	-	-
male	F=0.123, p=0.885	F=2.834, p=0.060	F=0.542, p=0.582	F=2.019, p=0.134	F=2.933, p=0.055	F=0.654, p=0.521	F=0.531, p=0.588	F=0.045, p=0.956	F=0.350, p=0.705	F=0.226, p=0.798
LSD post-hoc	-	-	-	-	-	-	-	-	-	-
female	F=1.374, p=0.257	F=0.498, p=0.609	F=0.624, p=0.537	F=1.066, p=0.347	F=0.349, p=0.706	F=0.964, p=0.384	F=0.610, p=0.544	F=1.480, p=0.231	F=2.478, p=0.087	**F=4.226, p=0.016**
LSD post-hoc	-	-	-	-	-	-	-	-	-	**HC>CS, p=0.023**
	-	-	-	-	-	-	-	-	-	**HC>CD, p=0.020**

Supplementary Table S4. Summary of results from Pearson's Correlations performed to determine interactions between fractional anisotropy status and methylation status, in all subjects, healthy controls, schizophrenia subjects, and male and female healthy control and schizophrenia subjects individually. Significant correlations shown in bold.

		**CpG1**	**CpG2**	**CpG3**	**CpG4**	**CpG5**	**CpG6**	**CpG7**	**CpG8**	**CpG9**	**CpG10**
**Fractional Anisotropy**	***all subjects***	r=0.009, p=0.899	r=-0.033, p=0.617	r=-0.037, p=0.573	r=0.007, p=0.573	r=0.029, p=0.651	r=-0.040, p=0.538	r=-0.045, p=0.482	r=-0.084, p=0.193	r=0.053, p=0.410	r=0.008, p=0.902
	healthy control	r=0.051, p=0.663	r=-0.150, p=0.168	r=0.020, p=0.850	r=0.083, p=0.442	r=0.014, p=0.894	r=-0.137, p=0.201	r=-0.140, p=0.185	r=-0.118, p=0.265	r=0.100, p=0.344	r=-0.040, p=0.706
	schizophrenia	r=0.003, p=0.972	r=0.035, p=0.673	r=-0.079, p=0.335	r=-0.025, p=0.765	r=-0.002, p=0.978	r=0.023, p=0.773	r=-0.019, p=0.816	r=-0.035, p=0.668	r=0.046, p=0.572	r=0.038, p=0.641
	male healthy control	r=0.063, p=0.654	r=-0.092, p=0.476	r=-0.004, p=0.973	r=0.081, p=0.527	r=-0.018, p=0.888	**r=-0.254, p=0.041**	r=-0.097, p=0.437	r=-0.108, p=0.389	r=0.019, p=0.879	r=-0.087, p=0.488
	female healthy control	r=0.011, p=0.963	r=-0.359, p=0.085	r=0.112, p=0.593	r=0.106, p=0.621	r=0.172, p=0.412	r=0.375, p=0.071	r=-0.308, p=0.134	r=-0.165, p=0.430	**r=0.423, p=0.035**	r=0.181, p=0.387
	male schizophrenia	r=-0.005, p=0.962	r=0.018, p=0.853	r=-0.068, p=0.487	r=-0.039, p=0.693	r=0.028, p=0.772	r=-0.051, p=0.601	r=-0.085, p=0.378	r=0.012, p=0.900	r=0.100, p=0.300	r=0.021, p=0.827
	female schizophrenia	r=0.021, p=0.903	r=0.028, p=0.856	r=-0.138, p=0.372	r=0.015, p=0.926	r=-0.097, p=0.528	r=0.144, p=0.344	r=0.137, p=0.369	r=-0.212, p=0.172	r=-0.084, p=0.585	r=0.076, p=0.620

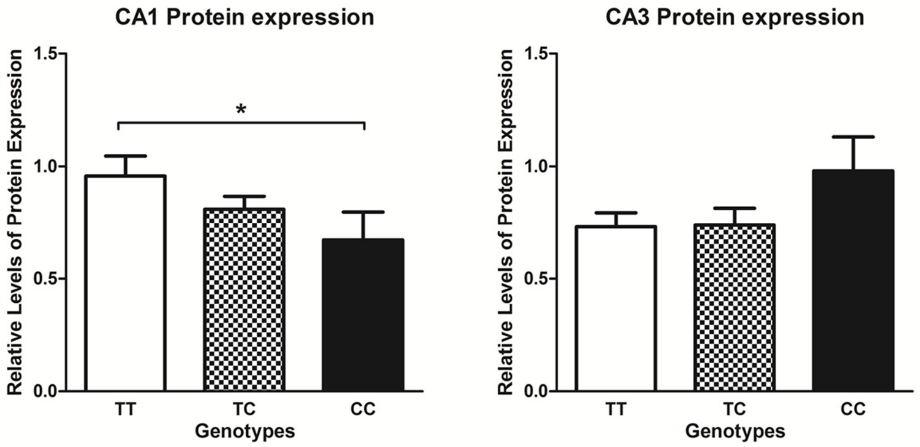

Supplementary Figure S1: Relative levels of Lingo-1 protein expression in the CA1 and CA3 hippocampal regions in relation to genotype. The major TT genotype seems to have a regionally specific role in regulating protein expression in the hippocampus, with the TT genotype resulting in significantly increased levels of Lingo-1 protein expression in the CA1 compared to the minor CC genotype ($p=0.022$). In the CA3 hippocampal region, this is the opposite, although not reaching statistical significance ($p=0.100$).
